# Unwinding of Continuous Medicaid Coverage Among Patients at Community Health Centers

**DOI:** 10.1001/jamahealthforum.2023.4622

**Published:** 2024-01-05

**Authors:** Wyatt P. Bensken, Siran M. Koroukian, Brenda M. McGrath, Philip M. Alberti, Erika K. Cottrell, Marion R. Sills

**Affiliations:** 1 Department of Research, OCHIN, Portland, Oregon; 2 Quantitative Sciences Core, OCHIN, Portland, Oregon; 3 Social Policy Program, OCHIN, Portland, Oregon; 4 Case Western Reserve University, Cleveland, Ohio; 5 AAMC Center for Health Justice, Association of American Medical Colleges, Washington, DC; 6 Oregon Health and Science University, Portland, Oregon; 7 University of Colorado, Anschutz Medical Campus, Aurora, Colorado

## Abstract

This retrospective cohort study uses data from the Accelerating Data Value Across a National Community Health Center Network to assess patterns of Medicaid disenrollment during the first 6 months after the end of continuous enrollment.

## Introduction

The US Families First Coronavirus Response Act of the COVID-19 pandemic required states to provide continuous Medicaid coverage to receive additional federal funding.^1^ In April 2023, the Consolidated Appropriations Act required states to resume standard redeterminations,^2^ with large initial decreases in Medicaid enrollment.^3^ We studied a large national network of community health centers to assess Medicaid disenrollment during the first 6 months after the end of continuous enrollment.

## Methods

This retrospective cohort study used data from the Accelerating Data Value Across a National Community Health Center Network^4^ that included real-time electronic health record data from a nationwide network of community health care organizations supported on OCHIN’s single instance of the Epic electronic health record.^5^ This study, including a waiver of informed consent, was approved by the Advarra Institutional Review Board and followed the STROBE reporting guideline.

The cohort included adult patients 18 years and older with an ambulatory or telehealth visit during continuous enrollment (April 1, 2020, to March 31, 2023) and after the provisions ended (April 1, 2023, to September 30, 2023) (eFigure in Supplement 1). These visits occurred at 1114 clinics within 158 health care organizations in 28 states.

The primary outcome was being disenrolled from Medicaid, defined as being Medicaid insured at the latest visit in the continuous enrollment period and uninsured at any visit in the post–continuous enrollment period. Our main independent variables were race and ethnicity and active comorbid conditions. To adjust for other factors associated with the primary outcome, the covariates sex, age at last visit during continuous enrollment, federal poverty level (FPL) percentage of income, state of residence, and the number of encounters (divided into quartiles) during continuous enrollment were included. The categories of race, ethnicity, and sex are described in the eMethods in Supplement 1.

We used a multilevel logistic regression model, with a random intercept to account for patient clustering within states, to identify the association of the covariates with disenrollment from Medicaid. We report adjusted odds ratios, 95% CIs, and the intraclass correlation of the model. Data analyses were conducted in R.

## Results

Of the 575 170 patients who met inclusion criteria and were insured through Medicaid during continuous coverage, 16.7% (n = 96 189) were uninsured for at least 1 visit during the 6 months after continuous enrollment.

Disenrollment was higher among younger adults, American Indian and Alaska Native and Black or African American patients, those with a lower FPL percentage, those with higher utilization, and those with conditions including HIV/AIDS, mental health conditions, and substance use conditions (Table 1).

**Table 1.** Description of Study Population

Variable	Patients, No. (%)		
	Overall (N = 575 170)	Maintained Medicaid coverage (n = 478 981)	Disenrolled from Medicaid (n = 96 189)
Race and ethnicity^a^			
American Indian and Alaska Native	5390 (0.9)	3818 (70.8)	1572 (29.2)
Asian	40 158 (7.0)	36 468 (90.8)	3690 (9.2)
Black or African American	94 006 (16.3)	75 283 (80.1)	18 723 (19.9)
Hispanic or Latino	202 042 (35.1)	169 647 (84.0)	32 395 (16.0)
Multiple races	5544 (1.0)	4493 (81.0)	1051 (19.0)
Native Hawaiian and Other Pacific Islander	979 (0.2)	855 (87.3)	124 (12.7)
Unknown	43477 (7.6)	36540 (84.0)	6937 (16.0)
White	18 3574 (31.9)	151 877 (82.7)	31 697 (17.3)
Sex^b^			
Female	372 232 (64.7)	309 594 (83.2)	62 638 (16.8)
Male	202 530 (35.2)	169 067 (83.5)	33 463 (16.5)
Other/unknown	408 (0.1)	320 (78.4)	88 (21.6)
Age range, y			
18 to 29	142 436 (24.8)	118 908 (83.5)	23 528 (16.5)
30 to 39	123 386 (21.5)	100 687 (81.6)	22 699 (18.4)
40 to 49	101 230 (17.6)	82 964 (82.0)	18 266 (18.0)
50 to 64	173 680 (30.2)	146 482 (84.3)	27 198 (15.7)
≥65	34 438 (6.0)	29 940 (86.9)	4498 (13.1)
FPL percentage of income			
0	212 457 (36.9)	174 203 (82.0)	38 254 (18.0)
1 to 50	72 244 (12.6)	59 804 (82.8)	12 440 (17.2)
51 to 100	96 679 (16.8)	80 745 (83.5)	15 934 (16.5)
101 to 138	43 745 (7.6)	37 208 (85.1)	6537 (14.9)
≥139	57 612 (10.0)	48 771 (84.7)	8841 (15.3)
Missing	92 433 (16.1)	78 250 (84.7)	14 183 (15.3)
No. of encounters, during continuous enrollment			
Mean (SD)	12.98 (18.42)	11.50 (13.93)	20.38 (31.56)
Median (IQR)	8.00 (3.00-16.00)	7.00 (3.00-15.00)	11.00 (4.00-23.00)
Elixhauser Comorbidity Groups^c^			
HIV/AIDS	10 694 (1.9)	7484 (70.0)	3210 (30.0)
Autoimmune conditions	10 021 (1.7)	8313 (83.0)	1708 (17.0)
Blood loss conditions	11 370 (2.0)	9253 (81.4)	2117 (18.6)
Hematological conditions	63 805 (11.1)	52 293 (82.0)	11 512 (18.0)
Cancer	14 963 (2.6)	12 536 (83.8)	2427 (16.2)
Chronic lung disease	81 520 (14.2)	66 130 (81.1)	15 390 (18.9)
Cardiovascular diseases	24 441 (4.2)	20 006 (81.9)	4435 (18.1)
Diabetes	95 492 (16.6)	79 419 (83.2)	16 073 (16.8)
Hypertension	148 104 (25.7)	123 718 (83.5)	24 386 (16.5)
Kidney and liver diseases	48 110 (8.4)	38 841 (80.7)	9269 (19.3)
Mental health conditions	169 656 (29.5)	129 667 (76.4)	39 989 (23.6)
Neurological conditions	26 379 (4.6)	21 083 (79.9)	5296 (20.1)
Obesity	105 445 (18.3)	87 184 (82.7)	18 261 (17.3)
Substance use conditions	65 044 (11.3)	47 276 (72.7)	17 768 (27.3)
Thyroid conditions	38 937 (6.8)	32 662 (83.9)	6275 (16.1)
Vascular diseases	9646 (1.7)	7933 (82.2)	1713 (17.8)
Weight loss	7442 (1.3)	5921 (79.6)	1521 (20.4)
State			
AK	226 (0.0)	183 (81.0)	43 (19.0)
AL	4713 (0.8)	3715 (78.8)	998 (21.2)
AR	959 (0.2)	880 (91.8)	79 (8.2)
CA	254 596 (44.3)	219 166 (86.1)	35 430 (13.9)
CO	1251 (0.2)	1089 (87.1)	162 (12.9)
CT	9960 (1.7)	7788 (78.2)	2172 (21.8)
GA	1135 (0.2)	720 (63.4)	415 (36.6)
ID	11 485 (2.0)	9014 (78.5)	2471 (21.5)
IL	31 954 (5.6)	28 544 (89.3)	3410 (10.7)
IN	8808 (1.5)	6697 (76.0)	2111 (24.0)
LA	202 (0.0)	135 (66.8)	67 (33.2)
MA	51 119 (8.9)	40 870 (80.0)	10 249 (20.0)
MD	139 (0.0)	59 (42.4)	80 (57.6)
ME	1415 (0.2)	1268 (89.6)	147 (10.4)
MN	6306 (1.1)	5161 (81.8)	1145 (18.2)
MO	487 (0.1)	378 (77.6)	109 (22.4)
MT	1795 (0.3)	1453 (80.9)	342 (19.1)
NC	10 225 (1.8)	9035 (88.4)	1190 (11.6)
NJ	8882 (1.5)	7027 (79.1)	1855 (20.9)
NY	5204 (0.9)	3344 (64.3)	1860 (35.7)
OH	29 260 (5.1)	19 772 (67.6)	9488 (32.4)
OK	9669 (1.7)	8655 (89.5)	1014 (10.5)
OR	66 098 (11.5)	55 775 (84.4)	10 323 (15.6)
PA	2386 (0.4)	1669 (69.9)	717 (30.1)
SC	1318 (0.2)	1216 (92.3)	102 (7.7)
TX	2524 (0.4)	1870 (74.1)	654 (25.9)
WA	32 198 (5.6)	27 036 (84.0)	5162 (16.0)
WI	20 856 (3.6)	16 462 (78.9)	4394 (21.1)

Abbreviation: FPL, federal poverty level.

^a^ Further details on race and ethnicity reporting are available in the eMethods in Supplement 1.

^b^ Further details on sex reporting are available in the eMethods in Supplement 1.

^c^ The Elixhauser Comorbidity Groups were created from the *International Statistical Classification of Diseases and Related Health Problems, Tenth Revision (ICD-10)* Elixhauser comorbidities from the Agency for Healthcare Research and Quality.

In the adjusted analysis (Table 2), American Indian and Alaska Native, Black or African American, Hispanic or Latino, those who reported multiple races, and those whose race was unknown had higher odds of being disenrolled relative to White patients (Table 2). Those with a higher number of visits had higher odds of disenrollment (Table 2). Those with HIV/AIDS, mental health conditions, and substance use conditions had higher odds of being disenrolled. There was substantial variation across states and the intraclass correlation of the model indicates approximately 8.3% of the variation among those who were disenrolled could be attributed to state differences.

**Table 2.** AORs and 95% CIs for Being Disenrolled From Medicaid Coverage

Term	AOR (95% CI)
Race and ethnicity^a^	
American Indian and Alaska Native	2.17 (2.04-2.31)
Asian	0.68 (0.65-0.70)
Black or African American	1.13 (1.10-1.15)
Hispanic or Latino	1.19 (1.17-1.22)
Multiple races	1.09 (1.02-1.17)
Native Hawaiian and Other Pacific Islander	0.88 (0.73-1.06)
White	1 [Reference]
Unknown	1.10 (1.07-1.13)
Sex^b^	
Female	1 [Reference]
Male	0.96 (0.94-0.97)
Other/missing	1.10 (0.88-1.38)
Age range, y	
18 to 29	1.15 (1.11-1.19)
30 to 39	1.19 (1.14-1.23)
40 to 49	1.17 (1.13-1.21)
50 to 64	1.02 (0.99-1.06)
≥65	1 [Reference]
FPL percentage	
0	1.15 (1.12-1.19)
1 to 50	1.09 (1.06-1.13)
51 to 100	1.06 (1.03-1.10)
101 to 138	1 [Reference]
≥139	0.99 (0.95-1.02)
Missing	0.95 (0.92-0.98)
No. of encounters	
Q1	1 [Reference]
Q2	1.04 (1.02-1.07)
Q3	1.13 (1.11-1.16)
Q4	1.92 (1.87-1.96)
Elixhauser Comorbidity Groups	
AIDS	1.63 (1.56-1.71)
Autoimmune conditions	0.95 (0.90-1.00)
Blood loss conditions	0.98 (0.94-1.03)
Hematological conditions	0.98 (0.96-1.01)
Cancer	0.96 (0.91-1.00)
Chronic lung disease	0.97 (0.95-0.99)
Cardiovascular disease	1.04 (1.01-1.08)
Diabetes	1.05 (1.03-1.08)
Hypertension	0.95 (0.93-0.97)
Kidney and liver disease	1.02 (1.00-1.05)
Mental health conditions	1.44 (1.42-1.47)
Neurological conditions	1.01 (0.98-1.04)
Obesity	0.98 (0.97-1.00)
Substance misuse	1.54 (1.50-1.57)
Thyroid conditions	0.94 (0.91-0.97)
Vascular conditions	1.02 (0.97-1.08)
Weight loss	1.05 (0.99-1.12)

Abbreviations: AOR, adjusted odds ratio; FPL, federal poverty level; Q, quartile.

^a^ We compared unadjusted estimates for race and ethnicity to our adjusted estimates to consider how the factors we adjusted for changed the directionality or mediated the association between race/ethnicity and disenrollment. The findings were largely consistent with our adjusted results. Odds ratio: American Indian and Alaska Native: 2.15 (95% CI, 2.02-2.28); Asian: 0.54 (0.52-0.55); Black or African American: 1.04 (95% CI, 1.02-1.06); Hispanic or Latino: 1.05 (95% CI, 1.03-1.07); multiple races: 1.15 (95% CI, 1.08-1.23); Native Hawaiian and Other Pacific Islander: 0.76 (95% CI, 0.68-0.85); and unknown: 0.97 (95% CI, 0.95-1.00). Further details on race and ethnicity reporting are available in the eMethods in Supplement 1.

^b^ Further details on sex reporting are available in the eMethods in Supplement 1.

## Discussion

Among patients with visits to community-based health care organizations, those who were American Indian and Alaska Native, Black or African American, and Hispanic or Latino had higher odds of being disenrolled from Medicaid after continuous enrollment. Additional risk factors for Medicaid disenrollment included more frequent prior visits, and diagnoses of HIV/AIDS, mental health needs, or substance use conditions. These findings call attention to the need to identify and address the reasons for inequities in Medicaid disenrollment and to implement strategies^6^ to assist patients most likely to lose coverage.

A limitation is that our findings may not be generalizable to other patients insured through Medicaid who are not treated at community centers. Without eligibility data, we could not differentiate between Medicaid dropout and loss of eligibility.

## Notes

Supplement 1.
eMethods
**eFigure.** Study Inclusion Flow Chart
eReferences

Supplement 2.
Data Sharing Statement
