# Supplementary material for: Unwinding of Continuous Medicaid Coverage Among Patients at Community Health Centers
Source: JAMA Health Forum. 2024 Jan 5;5(1):e234622. doi: 10.1001/jamahealthforum.2023.4622 (PMC10770764; doi:10.1001/jamahealthforum.2023.4622)
Supplement: Supplement 1. — eMethods eFigure. Study Inclusion Flow Chart eReferences [file jamahealthforum-e234622-s001.pdf]

Supplemental Online Content

Bensken WP, Koroukian SM, McGrath BM, Alberti PM, Cottrell EK, Sills MR. Unwinding of continuous Medicaid coverage among patients at community health centers. *JAMA Health Forum.* 2024;5(1):e234622. doi:10.1001/jamahealthforum.2023.4622

eMethods

eFigure 1. Study Inclusion Flow Chart

eReferences

This supplemental material has been provided by the authors to give readers additional information about their work.

© 2024 Bensken et al. *JAMA Health Forum*.

eMethods

The CHCs in this study come from the OCHIN network. All members of the OCHIN network use a single instance of Epic© that is centrally managed by OCHIN. Given this, OCHIN has access to clinical data from every visit that a patient has to any OCHIN member. Community Health Centers (CHCs) play a vital role in providing primary care to low-income, uninsured, Medicaid, and other marginalized populations.[1-3] CHCs reduce barriers to care including cost (i.e., serving all patients regardless of ability to pay), distance, and cultural competency, and are often the most readily accessed health care for minoritized populations.[4-6] CHCs have also been shown to improve outcomes, including bridging patients to specialty care and reducing acute care utilization, across a wide variety of conditions (e.g., hypertension, diabetes, and cancer).[5,7-18]

Race/ethnicity in this manuscript is a variable that we created from reported "Hispanic" and "Race" variables in the PCORnet common data model based on Office of Management and Budget (OMB) standards. Hispanic, which we renamed ethnicity, is defined as "A person of Cuban, Mexican, Puerto Rican, South or Central American, or other Spanish culture or origin, regardless of race." Possible levels of this were: yes, no, refuse to answer, no information, unknown, and other. The race variable includes the following levels: American Indian or Alaska Native, Asian, Black or African American, Native Hawaiian or Other Pacific Islander, White, Multiple races, Refuse to answer, No information, Unknown, and Other. The categorical definitions are as follows: American Indian or Alaska Native: A person having origins in any of the original peoples of North and South America (including Central America), and who maintains tribal affiliation or community attachment. Asian: A person having origins in any of the original peoples of the Far East, Southeast Asia, or the Indian subcontinent including, for example, Cambodia, China, India, Japan, Korea, Malaysia, Pakistan, the Philippine Islands, Thailand, and Vietnam. Black or African American: A person having origins in any of the black racial groups of Africa. Native Hawaiian or Other Pacific Islander: A person having origins in any of the original peoples of Hawaii, Guam, Samoa, or other Pacific Islands. White: A person having origins in any of the original peoples of Europe, the Middle East, or North African. Consistent with recent proposals to collapse race and ethnicity to a single category, we created a new "race/ethnicity" variable. Any patient that reported "Yes" for Hispanic was classified as "Hispanic" while those who reported "No" were classified as their reported race. Due to small cell sizes, we had to create one category of Other/Missing/Unknown. The final levels were: American Indian and Alaska Native, Asian, Black or African American, Hispanic or Latino, Multiple races, Native Hawaiian or Other Pacific Islander, /Unknown, and White. Due to small counts (< 20) "Other" was removed.

Sex was identified from the demographic information of the patient's electronic health record. This variable was defined as "sex assigned at birth" with possible levels of: ambiguous, female, male, no information, and unknown. Due to small cell size count we collapsed these levels to female, male, and other/unknown. Race, ethnicity, and sex are most likely completed by the patient upon enrollment at their first visit, however there may instances where others (e.g., a health care proxy) complete this information. It is unknown to us exactly who reports this information. Finally, we extracted FPL data associated with the most recent encounter in the post continuous enrollment period. FPLs of 0 were due to a reported income of 0, while a missing FPL was due to missing income.

© 2024 Bensken et al. *JAMA Health Forum*.

eFigure 1. Study Inclusion Flow Chart

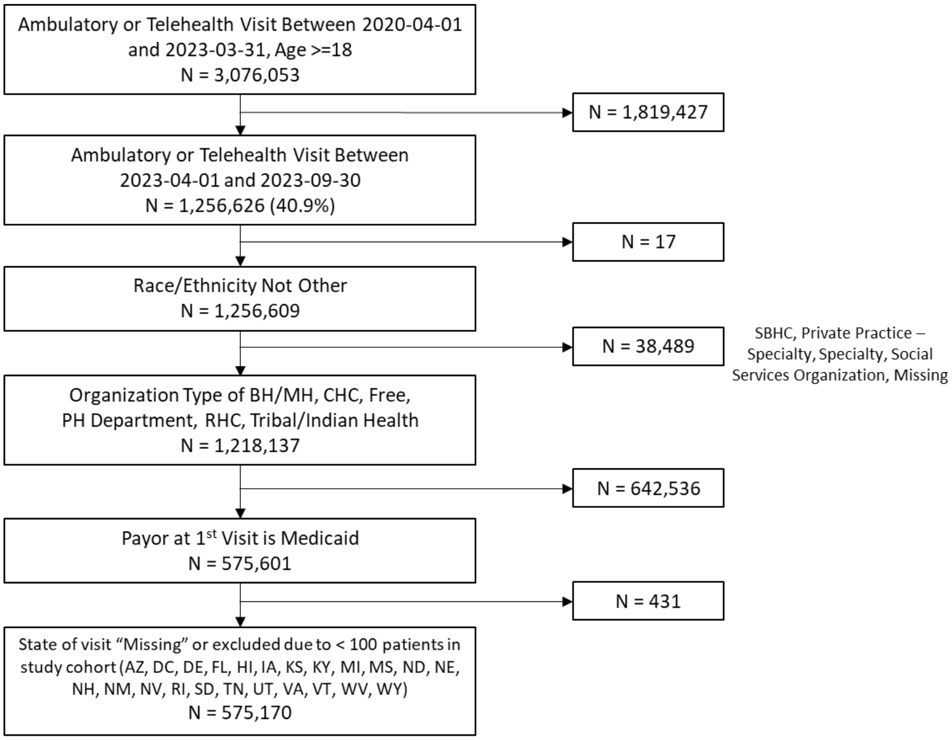

BH/MH: Behavior Health / Mental Health; CHC: Community Health Center, PH: Public Health Department; RHC: Rural Health Clinic; SBHC: School-based Health Center

© 2024 Bensken et al. *JAMA Health Forum*.

eReferences

1. Hadley J, Cunningham P. Availability of safety net providers and access to care of uninsured persons. *Health Serv Res.* 2004;39(5):1527-1546.

2. Epstein AJ. The role of public clinics in preventable hospitalizations among vulnerable populations. *Health Serv Res.* 2001;36(2):405-420.

3. Shi L, Stevens GD. The role of community health centers in delivering primary care to the underserved: experiences of the uninsured and Medicaid insured. *J Ambul Care Manage.* 2007;30(2):159-170.

4. Huguet N, Valenzuela S, Marino M, et al. Following Uninsured Patients Through Medicaid Expansion: Ambulatory Care Use and Diagnosed Conditions. *Ann Fam Med.* 2019;17(4):336-344.

5. Hoopes M, Schmidt T, Huguet N, et al. Identifying and characterizing cancer survivors in the US primary care safety net. *Cancer.* 2019;125(19):3448-3456.

6. National Association of Community Health Centers. Community Health Center Chartbook. 2020; https://www.nachc.org/wp-content/uploads/2020/01/Chartbook-2020-Final.pdf. Accessed September 22, 2020.

7. Gold R, DeVoe J, Shah A, Chauvie S. Insurance continuity and receipt of diabetes preventive care in a network of federally qualified health centers. *Med Care.* 2009;47(4):431-439.

8. Angier H, Huguet N, Marino M, et al. Observational study protocol for evaluating control of hypertension and the effects of social determinants. *BMJ Open.* 2019;9(3):e025975.

9. Blackburn BE, Marino M, Schmidt T, et al. Preventive service utilization among low-income cancer survivors. *J Cancer Surviv.* 2022;16(5):1047-1054.

10. Cottrell EK, O'Malley JP, Dambrun K, et al. The Impact of Social and Clinical Complexity on Diabetes Control Measures. *J Am Board Fam Med.* 2020;33(4):600-610.

11. Devoe JE, Gold R, McIntire P, Puro J, Chauvie S, Gallia CA. Electronic health records vs Medicaid claims: completeness of diabetes preventive care data in community health centers. *Ann Fam Med.* 2011;9(4):351-358.

12. Donovan J, Cottrell EK, Hoopes M, et al. Adjusting for Patient Economic/Access Issues in a Hypertension Quality Measure. *Am J Prev Med.* 2022;63(5):734-742.

13. Huguet N, Larson A, Angier H, et al. Rates of Undiagnosed Hypertension and Diagnosed Hypertension Without Anti-hypertensive Medication Following the Affordable Care Act. *Am J Hypertens.* 2021;34(9):989-998.

14. Shohet C, Yelloly J, Bingham P, Lyratzopoulos G. The association between the quality of epilepsy management in primary care, general practice population deprivation status and epilepsy-related emergency hospitalisations. *Seizure.* 2007;16(4):351-355.

15. Quinones AR, Valenzuela SH, Huguet N, et al. Prevalent Multimorbidity Combinations Among Middle-Aged and Older Adults Seen in Community Health Centers. *J Gen Intern Med.* 2022;37(14):3545-3553.

© 2024 Bensken et al. *JAMA Health Forum.*

16. Gusmano MK, Fairbrother G, Park H. Exploring the limits of the safety net: community health centers and care for the uninsured. *Health Aff (Millwood).* 2002;21(6):188-194.

17. Cook NL, Hicks LS, O'Malley AJ, Keegan T, Guadagnoli E, Landon BE. Access to specialty care and medical services in community health centers. *Health Aff (Millwood).* 2007;26(5):1459-1468.

18. Timbie JW, Kranz AM, Mahmud A, Damberg CL. Specialty care access for Medicaid enrollees in expansion states. *Am J Manag Care.* 2019;25(3):e83-e87.

© 2024 Bensken et al. *JAMA Health Forum*.
