# Supplementary material for: Unwinding of Continuous Medicaid Coverage Among Patients at Community Health Centers
Source: JAMA Health Forum. 2024 Jan 5;5(1):e234622. doi: 10.1001/jamahealthforum.2023.4622 (PMC10770764; doi:10.1001/jamahealthforum.2023.4622)
Supplement: Supplement 2. — Data Sharing Statement [file jamahealthforum-e234622-s002.pdf]

Data Sharing Statement

Bensken. Unwinding of Continuous Medicaid Coverage Among Patients at Community Health Centers. *JAMA Health Forum*. Published January 05, 2024.
doi:10.1001/jamahealthforum.2023.4622

Data
Data available: No
